# Platelet-rich fibrin for rehydration and pre-vascularization of an acellular, collagen membrane of porcine origin

**DOI:** 10.1007/s00784-023-05485-2

**Published:** 2024-01-16

**Authors:** Saskia-Vanessa Schröger, Sebastian Blatt, Kawe Sagheb, Bilal Al-Nawas, Peer W. Kämmerer, Keyvan Sagheb

**Affiliations:** 1 Department of Oral and Maxillofacial Surgery—Plastic Operations, University Medical Center Mainz, Augustusplatz 2, 55131 Mainz, Germany; 2 Department of Prosthodontics University Medical Center Mainz, Mainz, Germany

**Keywords:** Platelet-rich fibrin, Collagen matrix, In ovo cam assay, Angiogenesis, pH value, Guided bone regeneration

## Abstract

**Objectives:**

Pre-vascularization of the collagen membranes with autologous platelet concentrates is a standard procedure in oral and maxillofacial surgery. This study analyzed the possible interaction of an acellular collagen membrane of porcine origin (NM) with platelet-rich fibrin (PRF) regarding its rehydration protocol with differences in pH values and effect on angiogenesis.

**Materials and methods:**

NM was analyzed alone and combined with solid PRF by plotting or co-culturing with injectable PRF. Different media (venous blood, buffer solution with a fixed pH value of 7, saline solution, and injectable PRF) were used to analyze the influence on pH value during rehydration. Chorion allantois membrane assay (CAM) was applied to check pro-angiogenic effects after 24, 48, and 72 h, followed by immunohistochemical analysis.

**Results:**

Rehydration in injectable PRF showed acidity over time (*p* < 0.05). A definite pro-angiogenic effect of NM alone was found regarding neo-vessel formation supported by the respective light microscopically analysis without significant differences to PRF alone (*p* > 0.005). This pro-angiogenic effect could not be exaggerated when NM was combined with liquid/solid PRF (each *p* > 0.005).

**Conclusions:**

Rehydration with liquid PRF of the collagen membrane results in acidity compared to a saline solution or patient’s blood. The significant pro-angiogenic potential of the membrane alone resulted in enhanced neo-vessel formation that could not be optimized with the addition of PRF.

**Clinical relevance statement:**

Using injectable PRF for rehydration protocol of the collagen membrane leads to acidosis that can ultimately optimize wound healing. Differences in the physio-mechanical interplay of collagen matrices and autologous platelet concentrates must result in clinical algorithms if pre-vascularization can maximize outcomes.

## Introduction

The principles of guided bone or guided tissue regeneration (GBR/GTR) and the usage of membranes go back to experimental studies on the regeneration potential of periodontal tissues in the 1970s and 80s [1]. GBR and GTR techniques can be applied before, during, or after implantation. They are essential in stabilizing the alveolar ridge height and width and the surrounding soft tissue after tooth extraction [2]. Autologous, allogeneic, xenogenic, or alloplastic biomaterials are used. In addition, mucosal transplants or resorbable or non-resorbable barrier membranes can be used to ensure ideal biological conditions for further treatment procedures [3–5].

Theoretically, the membrane can act like an occlusive barrier and create a separated space, facilitating ingrowth only by cells from the periodontal ligament or the alveolar bone. They should exclude nonosteogenic tissue and stabilize the grafted defect [6, 7]. The stability and placeholder function provided by the barrier membrane are much more essential and contribute significantly to the success of regeneration. It prevents soft tissue from collapsing into the defect and accumulates growth factors [8]. Therefore, the membranes must have a particular strength and architecture and the ability to support proliferation and vascularisation to improve tissue regeneration [9].

First, non-resorbable products based on Teflon were designed [6]. Still, in clinical use, they are rigid, requiring additional mini-screws or pins for stabilization, and must be removed in a follow-up procedure before definitive restoration. In addition, gingival recessions and membrane exposure can occur during the healing process, resulting in wound infection and subsequent early removal of the membrane [5, 10]. Here, biodegradable materials improved the clinical outcome without requiring a second intervention to remove the foreign material. Furthermore, they are more cost-efficient and show lower morbidity [5, 6, 10]. There are different resorbable membranes available, but predominantly collagen membranes, which are made from collagen type I or a combination of type I and III collagen of bovine or porcine origin (dermis, pericardium, or peritoneum), are used [11–13].

The collagen membranes offer similar clinical outcomes to non-resorbable membranes, especially in horizontal defects, and lower rates of complications and play an essential role in promoting bone regeneration by influencing vascularization [14]. In this context, however, the membranes can also act as a barrier to newly sprouting vessels and possibly lack or delay the blood supply, which remains a significant clinical limitation [10]. Another disadvantage is poor volume stability due to rapid degradation [15]. Therefore, various physical, chemical, or enzymatic techniques have been investigated to modify and improve the mechanical properties of the membranes and their regenerative capacity. For example, a combination of magnesium meshes, proteins, and growth factor nanoparticles seems feasible. This approach is hampered by restrictive regulations. A “clinical” modification or biofunctionalization of collagen matrices uses autologous platelet concentrates, the subject of the recent literature discussion [16, 17].

There is sufficient evidence that platelets are essential in primary hemostasis and bone and soft tissue healing. They are reservoirs of growth factors and release high concentrations of biologically active proteins that stimulate the recruitment of cells from the surrounding host tissue [18, 19]. Autologous platelet concentrates (APC) have been used to optimize regeneration for decades. Since the first description of platelet-rich human plasma, different techniques have emerged [20]. The second generation of APC, such as platelet-rich fibrin (PRF), are easily accessible, available, instantly ready to use, and cost-efficient. Using different centrifugation protocols, it is now possible to produce liquid, injectable, and more stable forms/membranes of autologous platelet concentrates. The injectable liquid form demonstrates the highest concentration of leukocytes and platelets compared to the more solid PRF membrane [18, 19]. Introduced in 2001 by Choukroun et al., PRF is now widely used in oral and maxillofacial surgery and is considered an essential component of craniofacial regeneration treatment concepts [20, 21]. It is used, for example, in alveolar management after extraction/socket preservation, bone augmentation, regeneration, sinus floor elevation, or therapy of gingival and periodontal bone defects [22–25]. Numerous in vivo and in vitro studies have shown that both PRF variants can positively influence angio- and vasculogenesis when combined with biomaterials such as bone substitute materials or collagen matrices and can be used for pre-vascularization and biofunctionalization of these materials. This approach broadens the indication for clinical use of the respective materials [22, 23, 26–28]. In a recent study, the effects of PRF on the rehydration process were investigated, showing that PRF represents a promising alternative for collagen membrane rehydration compared with blood or saline solution to enhance their vascularization [29]. It is well known that the rehydration protocol significantly impacts the biomechanical properties of the membranes [30]. In addition, depending on the incubation medium chosen, changes in pH can indirectly influence vascularisation after the rehydration process. This is of great interest considering the pH value’s explicit role in wound healing sites [29, 31].

This study analyzed the possible interaction of an acellular dermal matrix (NM) of porcine origin with different forms of platelet-rich fibrin regarding the rehydration protocol and its pro-angiogeneic effect. The hypothesis is that rehydration of NM with PRF leads to favorable changes in pH value in comparison with blood or saline solution. Furthermore, it is anticipated that the combination of NM with liquid and solid PRF leads to an enhanced pro-angiogenic effect.

## Materials and methods

### Collagen membrane

NovoMatrix™ Reconstructive Tissue Matrix (NM; RTM; *Biohorizons, Birmingham/Allergan, Inc., USA; CAMLOG Vertriebs GmbH, Wimsheim, Germany*) is a porcine, acellular dermal collagen matrix that has a uniform thickness of 1 mm. The membrane is pre-hydrated in a patented aqueous phosphate-buffered solution containing matrix stabilizers. It is advised that rehydration should be done for at least 2 min, and the membrane can remain in the sterile solution for a maximum of 4 h before its use [32, 33]. The present study placed the membrane in different solutions for 5 min before use. During this time, the pH value was also measured.

### Platelet-rich fibrin protocol

PRF was processed as previously described [29], according to the protocol of Choukroun et al. [18]. Venous blood samples were taken from two healthy volunteers who gave written consent. All procedures were conducted by the Declaration of Helsinki and approved by the Ethics Committee of Landesärztekammer Rhineland-Palatine (no. 2019-14705_1). Special vacutainer systems (10 ml; glass (A-PRF) and plastic tubes (I-PRF) without any coatings; Process for PRF, Nice, France) were used. The samples were directly centrifuged (A-PRF 1300 rpm for 8 min, i-PRF 700 rpm, 5 min relative centrifugal force 177* g* at a fixed angle rotor with a radius of 110 mm; Duo centrifuge, Process for PRF, Nice, France) in accordance with the respective manufacturer instructions [18, 34]. The solid PRF (A-PRF) was then pressed manually under sterile conditions with its corresponding PRF box (Process for PRF, Nice, France) for 60 s and cut into pieces 5 × 5 mm in size by using a sterile scalpel.

### Biofunctionalization of the membrane

For biofunctionalization, NM was pressed with the PRF scaffolds or co-incubated with 2 ml of the injectable PRF. The following samples were analyzed: native NM, solid PRF and NM, injectable PRF and NM, and PRF alone.

### Measurements of differences in pH value during rehydration

To analyze the influence on pH value during rehydration, the pre-hydrated NM was unpacked then put into a sterile basin and, according to the preparing instructions, rehydrated with saline solution for 5 min. The pH value was also measured during the rehydration process. The rehydrated membrane was afterward incubated for 20 min with the following media (5 ml): venous blood, buffer solution with a fixed pH value of 7, saline solution, and injectable PRF. The pH value was measured every 5 min with a glass pH electrode (pH-Meter CG840; Schott, Mainz, Germany). To avoid clotting of the venous blood, 2 ml of heparin was added (25,000 IU/5 ml solution; LEO Pharma GmbH, Neu-Isenburg, Germany). Before measuring, the pH electrode was calibrated with buffer solutions of pH values 4 and 7, and the mean pH values of the pure media were calculated to be able to exclude possible fluctuations of the pH value before adding the membrane. All experiments were done three times in triplicates (total *n* = 36).

### Chorion allantois membrane assay

As previously described, fertilized white Leghorn chicken eggs (LSL Rhein-Main, Dieburg, Germany) were incubated at 38 °C at constant humidity in a special incubator (Janeschitz, Hammelburg, Germany) [26]. On day 8 of embryological development, 8–10 ml of egg white was taken from the marked pole with a sterile syringe, and an oval 3 × 3 cm opening was cut into the surface with a sterile scissor. Pure NM, NM combined with solid or injectable PRF, and pure PRF membranes were applied to the chorion allantois membrane (CAM) under sterile conditions, close to the main vessels, keeping a safe distance from the embryo. For further incubation, the eggs were sealed again with parafilm and incubated as described above. After 24, 48, and 72 h, vascularization in a defined region of interest (ROI) of 500 × 500 µm was analyzed using a digital microscope at × 50 and × 100 magnification (VHX-1000; Keyence, Neu-Isenburg, Germany). These images were evaluated with an automatized software (IKOSA Prisma CAM Assay 3.1.0, KML Vision GmbH, Graz) to define the vessel area, length, thickness, and branching points. Afterward, samples were taken and embedded in Roti-Histofix 4.5% (Carl Roth GmbH + Co. KG, Karlsruhe, Germany) for further histological preparation. Finally, embryos were euthanized quickly after the experiments by severing the main vessels. All experiments were done three times in triplicates per investigated time point (total *n* = 108). All procedures were agreed with the 3Rs concept (the concept of reduction, refinement, and replacement of animal models). Since the chick embryo does not develop pain perception before the 17th day of incubation, no permission or approval from ethics committees is required [35].

### Histological preparation and staining

As described previously [29], samples were prepared for histological preparation and staining. Briefly, samples were fixed, cast, and embedded, and a histological section of 5 µm was made. For immunohistochemical staining, samples were deparaffined and placed 3 × 15 min in xylene and processed within hematoxylin–eosin staining (Merck, Darmstadt, Germany), anti-alpha smooth muscle actin antibody (1A4) (1:1000; Sigma-Aldrich, St. Louis, MO, USA), and immunofluorescence CD105 anti-chicken antibody (Biorbyt, Cambridge, England). The α-SMA staining specimens were dewaxed before proteins were unmasked in a steam cooker for 30 min. Next, samples were treated with a peroxidase (Dako, Jena, Germany) and a protein block (Dako, Jena, Germany) before adding the α-SMA antibody (diluted 1:1000). After 1 h, incubation marked polymer-HRP anti-mouse (Dako, Jena, Germany) was added for another 30 min. Before rehydration, the specimens were treated with DAB (Dako, Jena, Germany). For the CD 105 staining, specimens were dewaxed and treated with 0.1% Triton-X-100 (Sigma-Aldrich, St. Louis, USA) for 5 min. Blocking was performed by using PBS/BSA (Sigma-Aldrich, St. Louis, USA) and PBS/goat normal serum (Dako, Jena, Germany) before the samples were incubated with the CD 105 antibody (diluted 1:750) for 1 h, followed by incubation with the secondary antibody alpha rabbit 488 (diluted 1:100, Invitrogen, Carlsbad, USA) for an additional hour. Finally, cell nucleus staining was performed using DAPI (Thermo Fischer, Waltham, USA). The incubation of the last two steps was made in a dark environment due to the fluorescent characteristics of the staining [29]. Analysis was performed via Keyence Biorevo BZ-9000 light microscopy (KEYENCE, Neu-Isenburg, Germany), and the vessels were counted manually in the defined region of interest for HE-stained specimens. Immunohistochemically stained specimens were examined with the corresponding BZII-Viewer-Analyzer program (Brightfield HF and Phako, microscope position 2 Plan Apo Na.10; Keyence, Neu-Isenburg, Germany) as previously described [29]. All experiments were done three times in triplicates per investigated time point (total *n* = 108).

### Statistical analysis

Statistical analysis was performed with SPSS (version 27; IBM, Ehningen, Germany). The Kolmogorov–Smirnov and Shapiro–Wilk tests were applied to prove parametric terms. Without normal distribution, the Kruskal–Wallis test was used to identify significant differences within the individual groups over time. If differences were found, pairwise examinations followed using single-factor ANOVA, according to Kruskal–Wallis. To analyze the differences between the different groups, the Mann–Whitney *U* tests were used. The Friedman test was assessed to identify disparities within the measurements during 20 min for pH value analysis. According to the Kruskal–Wallis test, differences between the samples were also verified with a single-factor ANOVA. Boxplots were used for data illustration. *p-*values *p* < 0.05 were considered statistically significant.

## Results

### Interaction of PRF and collagen matrix influences the rehydration process

Pure saline solution showed a pH-mean value of 5.07 (± 0.66 standard deviation (SD)), pH 7 solution of 6.97 (± 0.013 SD), injectable PRF of 7.66 (± 0.15 SD) and blood of 7.52 (± 0.05 SD). NM was first placed in sodium chloride solution at room temperature as recommended by the manufacturer. The mean pH value in the initial rehydration process of the matrix was 5.76 (± 0.64 SD) after 5 min rehydration, 5.9 (± 0.62 SD) after 20 min, and 5.89 (± 0.624 SD) after another 20 min in saline solution. After insertion of the matrix, the pH 7 buffer solution showed a pH value of 7.03 (± 0.012 SD), injectable PRF 7.69 (± 0.09 SD), and blood 7.52 (± 0.05 SD). The pH values showed no significant difference during this period. After 5 min rehydration, a significant alkaline effect could be seen (5 min vs. 20 min, 0 min vs. 15 min, and 0 vs. 20 min, each *p* < 0.001) in saline solution. Incubation of the rehydrated NM with pH 7 solution and injectable PRF changed pH values significantly after 20 min (0 min vs. 20 min *p* < 0.02 and 5 min vs. 20 min. *p* < 0.05) and became acidic over time. In the blood, the pH values were constant and without significant differences.

### Interaction of PRF and the acellular collagen matrix does not significantly enhance neo-vessel formation in vivo

**Fig. 1:**
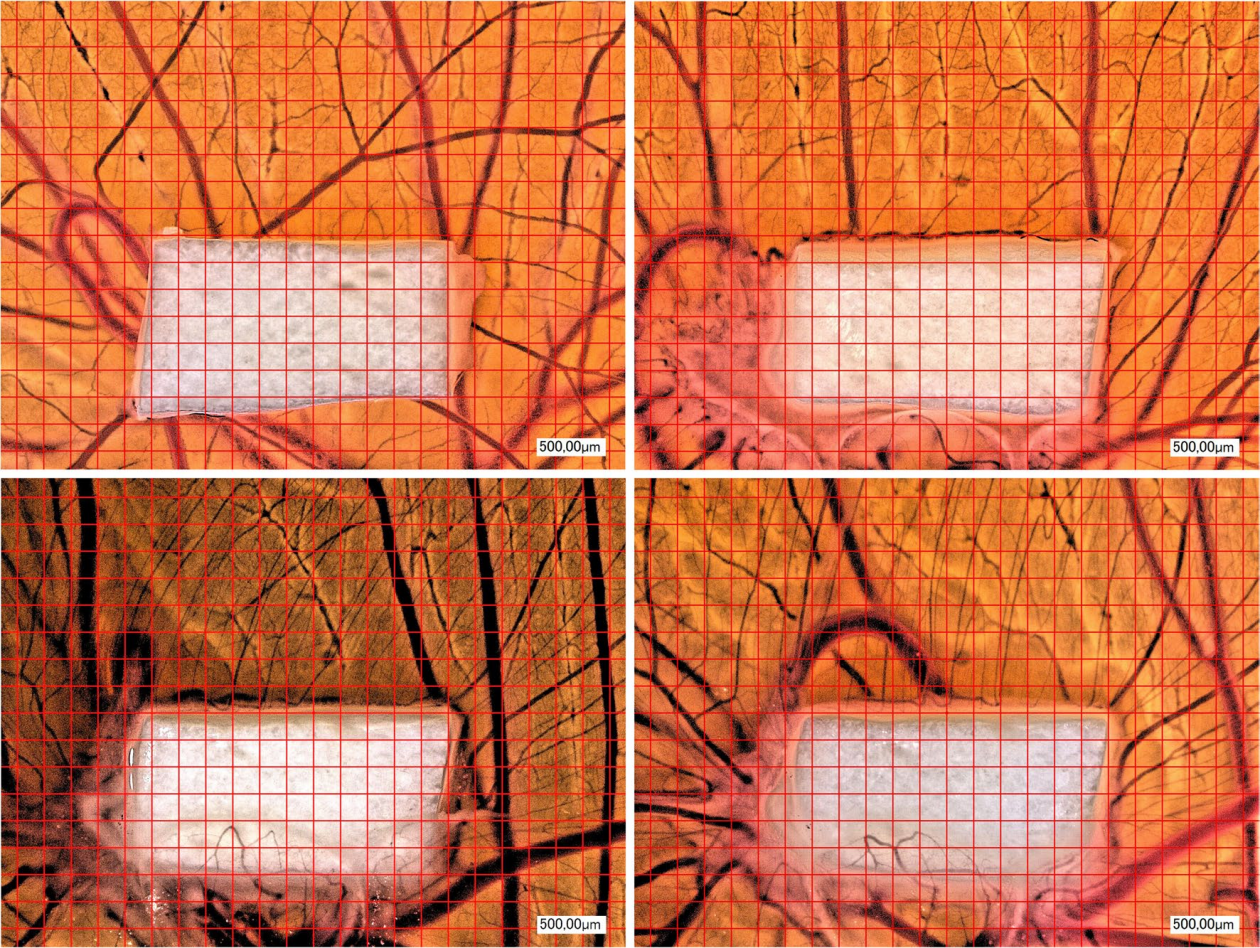
Example of microscopic analysis with pure NM (0 h, 24 h, 48 h, 72 h) within CAM assay. Magnification, 50-fold

**Fig. 2:**
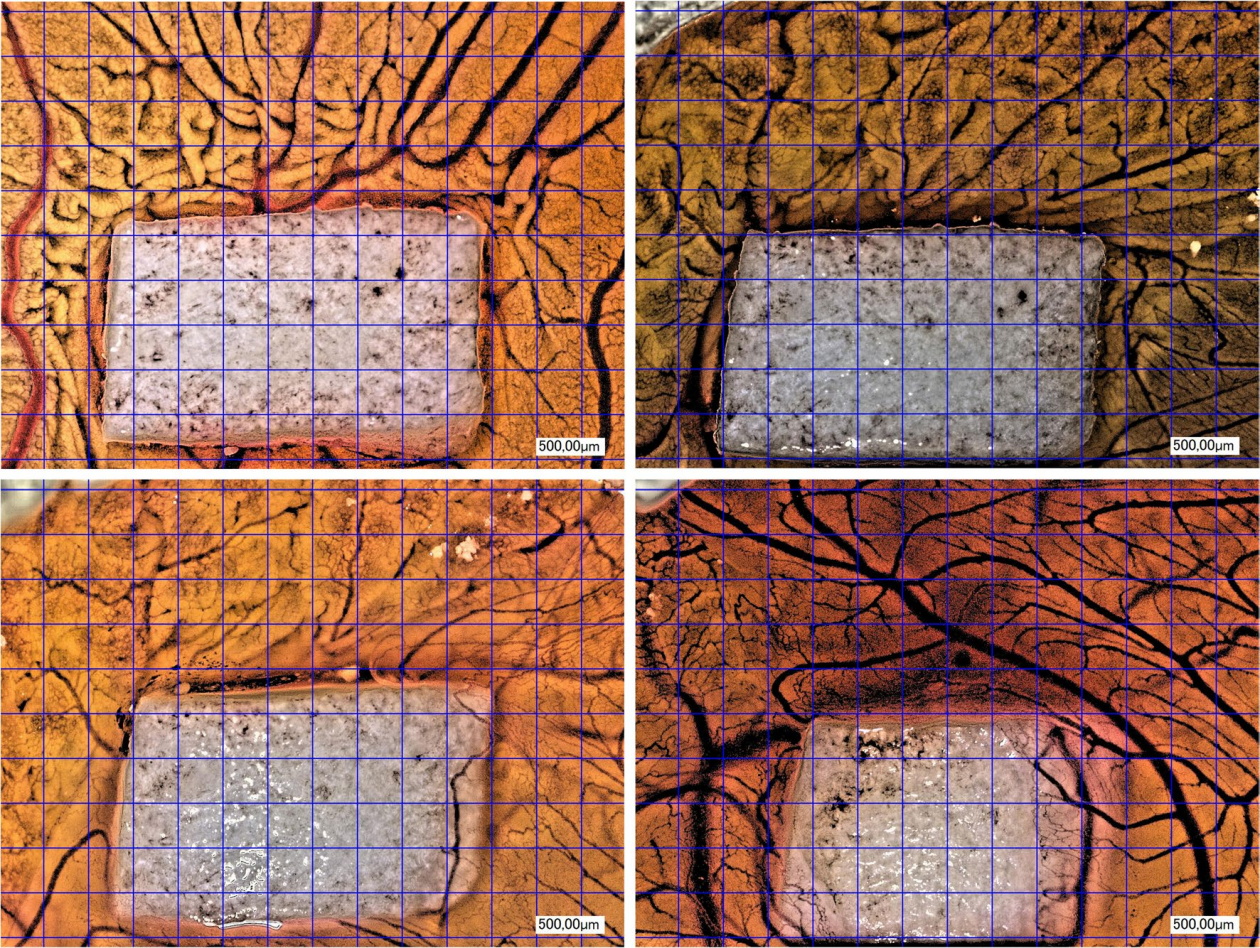
Example of microscopic analysis with a combination of NM + A-PRF (0 h, 24 h, 48 h, 72 h) within CAM assay. Magnification, 50-fold

**Fig. 3:**
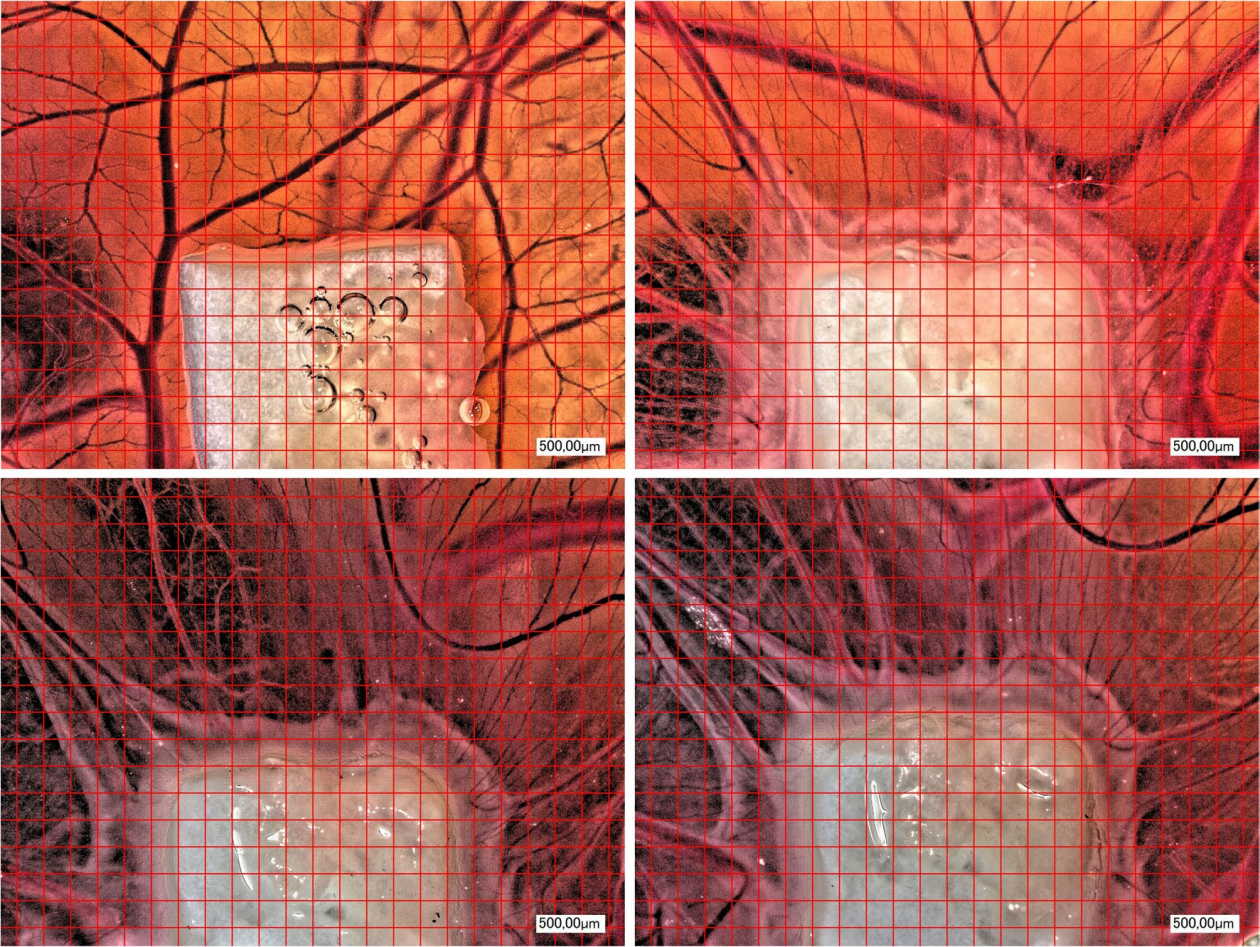
Example of microscopic analysis with a combination of NM + i-PRF (0 h, 24 h, 48 h, 72 h) within CAM assay. Magnification, 50-fold

**Fig. 4:**
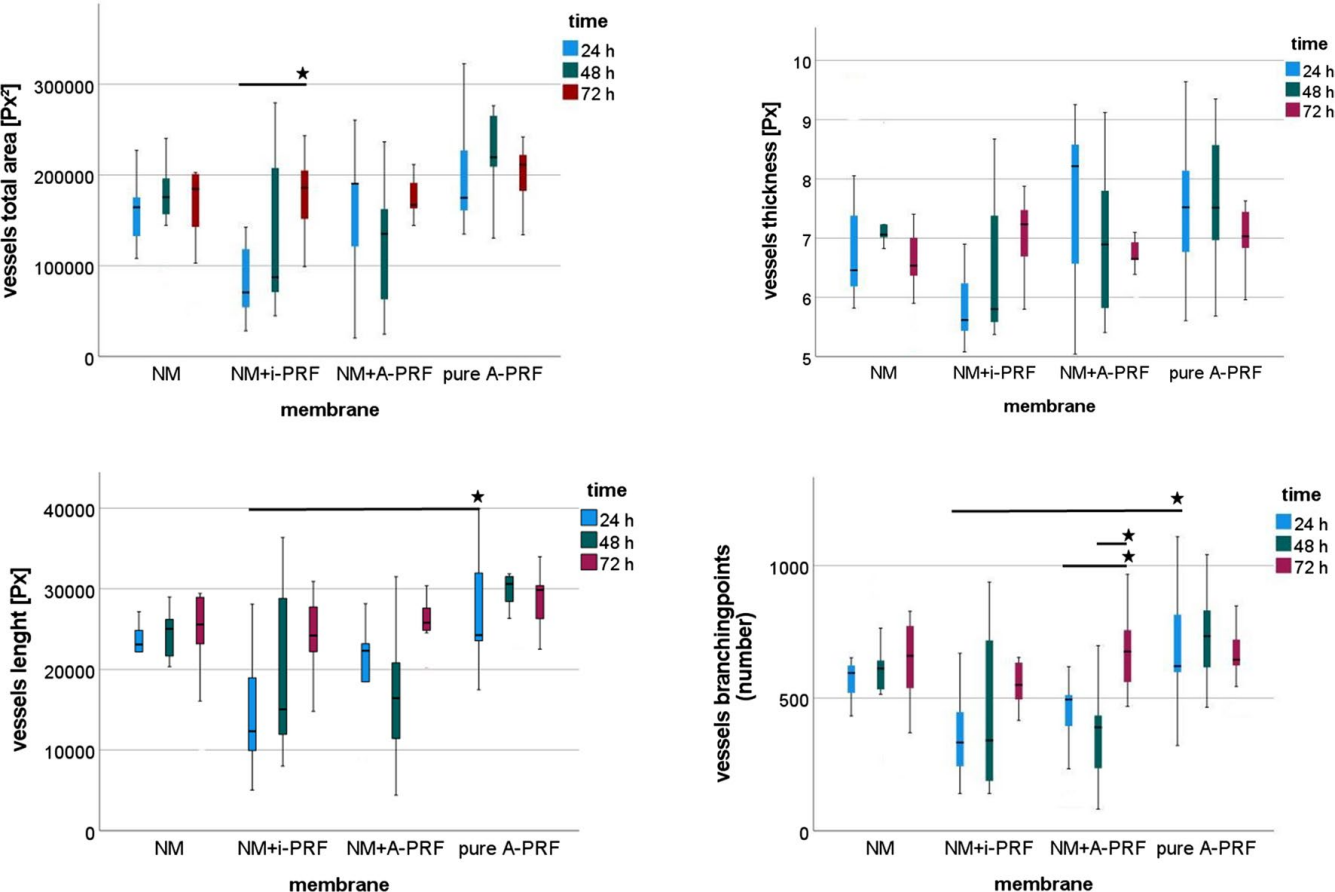
Light microscopical analysis of CAM-assay data with IKOSA® (vessels area in pixel^2^ [Px.^2^], length in pixels [Px], thickness in pixels [Px], and number of detected branching points) for the respective matrix (NM, NM + i-PRF, NM + A-PRF, pure A-PRF). *Statistically significant differences (*p* < 0.05; for details, please see the responding paragraph)

Comparing all groups (NM, NM + injectable PRF, NM + solid PRF, solid PRF alone) macroscopically, there was an increase in neo-vessel formation over time for all groups with a definite pro-angiogenic effect on the membrane itself (Figs. 1, 2, and 3). This observation was supported by the respective light microscopically analysis concerning total vessel area, vessel length, thickness, and branching points (Fig. 4). Here, NM with solid PRF showed increased branching points over time (24 h vs. 72 h *p* = 0.048 and 48 h vs. 72 h *p* = 0.003). NM + i-PRF showed a significantly increased total vessel area after 72 h compared to 24 h (*p* = 0.035). Comparing all groups at the different time points, NM did display high values for vessel area, length, and branching points without significant differences to PRF alone (*p* > 0.05). This pro-angiogenic effect could not be exaggerated with the combination of NM and solid or injectable PRF. Stable PRF alone enhanced neo-vessel formation with a significant increase in branching points (*p* = 0.047) and vessel length (*p* = 0.047) after 24 h compared to NM in combination with injectable PRF. No statistically significant difference existed between all samples at other investigated time points.

**Fig. 5:**
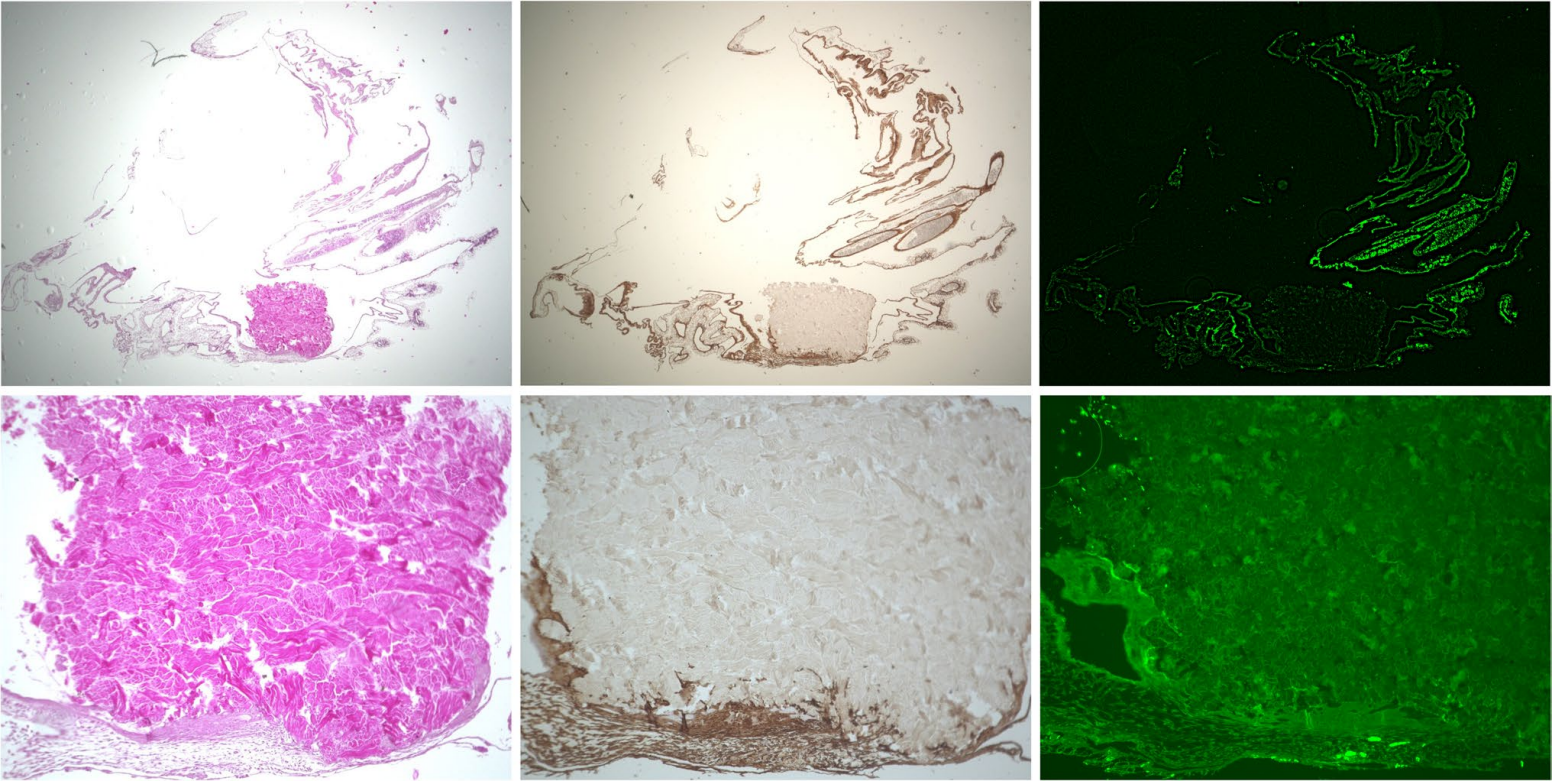
Example of a combination of NM with i-PRF. Immunohistochemical staining, left HE, middle SMA, right CD 105. Magnification, twofold and tenfold

**Fig. 6:**
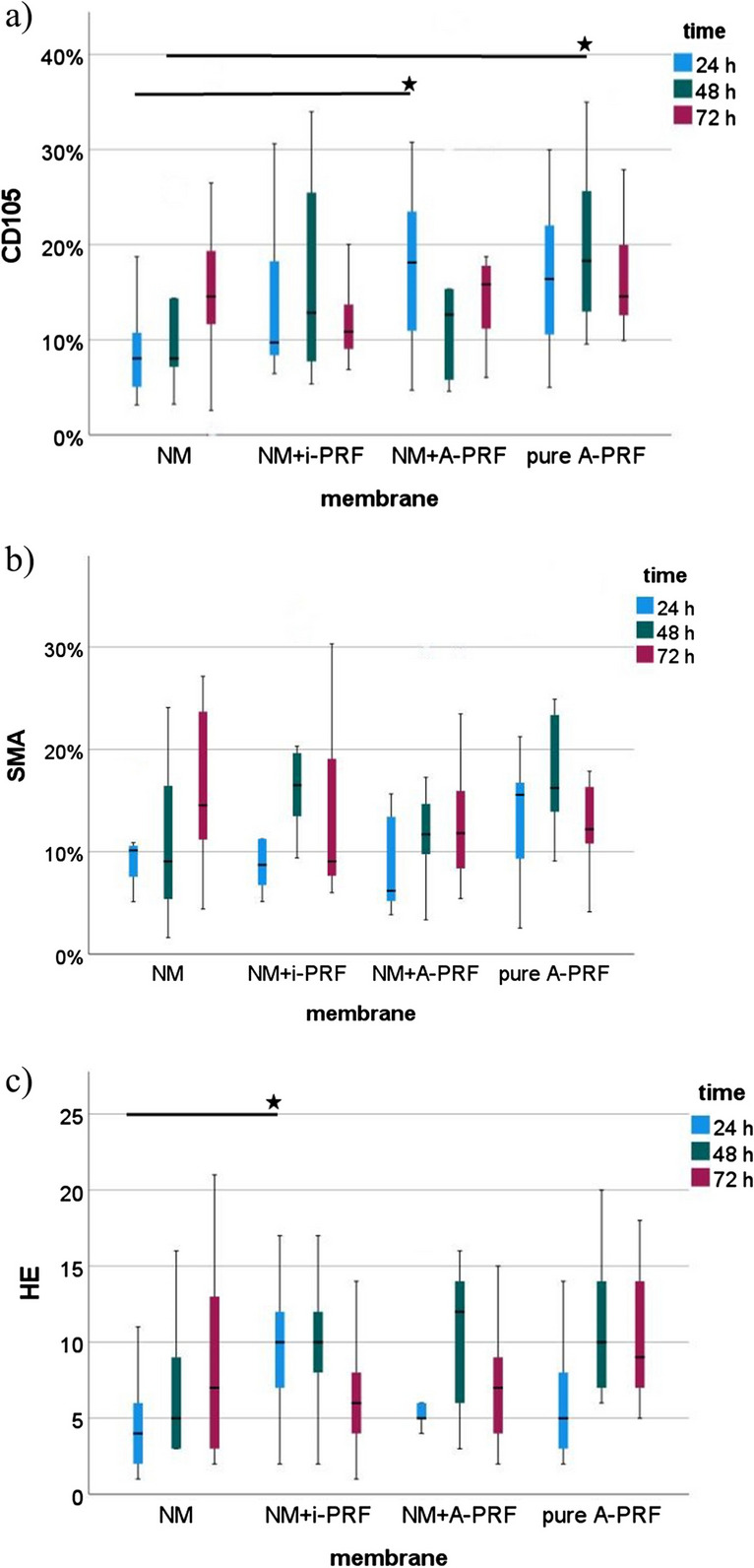
Analysis of immunohistochemical staining for the respective matrix (NM, NM + i-PRF, NM + A-PRF, pure A-PRF). **a** CD105. **b** SMA. **c** HE. *Statistically significant differences (*p* < 0.05; for details, please see the responding paragraph)

These observations could be confirmed immunohistochemically (Figs. 5 and 6). Descriptively, a vessel increase was seen in all stainings between 24 h and 72 h within the native NM group and the pre-vascularized NM with solid and injectable PRF between 24 h and 48 h. Although the combination of NM and solid PRF vs. native NM significantly increased vessels in the CD105 staining (*p* < 0.05) after 24 h, this effect was leveled over time. Contrary to light microscopic analysis, solid PRF alone showed a vessel increase between 24 h and 48 h in HE staining (*p* < 0.05) and increased vascularization in CD 105 staining after 48 h compared to NM. In the SMA staining, no significant differences between the groups to the respective time points could be found.

## Discussion

This study is the first to analyze the interaction between an acellular collagen membrane of porcine origin (NM) with solid and injectable autologous platelet concentrates and its implication for the rehydration process and pro-angiogenic potential. The presented results prove that injectable PRF for rehydration protocol of the collagen membrane is feasible and leads to favorable acidosis. Additionally, this study demonstrated for the first time that NM displays high pro-angiogenic characteristics that could not be exaggerated with the combination of NM and solid or injectable PRF.

With vague recommendations provided by the manufacturers regarding the rehydration medium and period, the appropriate rehydration protocol for the collagen membranes is still being discussed [36]. This contrasts pre-clinical data that demonstrate a direct influence on (pre)hydration protocol on direct and indirect cytotoxicity [37]. Additionally, rehydration affects pH values depending on the period and medium. This can directly regulate angiogenesis, collagen formation, macrophage activity, and neovascularization and ultimately influence tissue regeneration [29, 38, 39]. It is known that regeneration and wound healing are metabolically active processes, and pH values change in dependence on wound healing phases: a temporary acidosis leads to the impaired binding capacity of hemoglobin and subsequently increased oxygen release [39]. This favorable acidosis was achieved when NM was rehydrated with PRF instead of saline solution or blood. This follows a previous study where PRF alone was found initially alkaline but became acidic over time. The addition of collagen membranes enhanced this effect [29]. As a result, PRF seems more suitable for rehydration protocols of collagen membranes than saline solution.

Emerging evidence suggests that acellular collagen matrices may provide a viable alternative to autogenous soft tissue grafts in oral and maxillofacial regenerative procedures [36]. The clinical success relies heavily on its unique biomechanical and biochemical properties that mimic the biological and mechanical characteristics of the native extracellular matrix (ECM). Here, the matrix can be seen as a biological scaffold that supports the ingrowth and repopulation of fibroblasts and epithelium from surrounding tissue procedures [36]. Ideally, the GTR/GBR membrane should actively regulate wound/bone-repair-related cells (e.g., immune cells, endothelial cells, and stem cells) and stabilize the defect [8, 40]. However, with their occlusal characteristics, endothelial cell migration may also be impaired [26]. This way, vascularization is restricted. Due to different processing steps, collagen matrices are decellularized, deproteinized, and avascular and, therefore, heavily rely on neo-vascularization, which is indispensable for appropriate tissue regeneration, repair, and remodeling [26]. Pre-vascularization of these materials represents a solution to overcome this limitation. There is emerging evidence that autologous platelet concentrates such as PRF can ultimately lead to increased vascularization [26]. In this study, the evaluated parameters, vessel density, length, and branching points were not inferior for the membrane alone compared to solid PRF as the positive control. This was not the case in other studies where other collagen matrices displayed lower vessels and branching point levels compared to PRF alone [26, 29]. One may hypothesize a certain pro-angiogenic membrane potential that could only be partly exaggerated by biofunctionalization via PRF. At least for injectable PRF, there was no additional benefit seen. However, the analysis of the respective time points found a significantly increased total vessel area between 24 and 72 h. In contrast, the combination of stable PRF and the respective membrane led to a noteworthy but insignificant increase for the investigated parameters. Of note, branching points could be increased significantly over time when NM was combined with solid PRF. The results indicate that pre-vascularization of NM with PRF is feasible but does not result in higher vascularization features of the membrane.

The mechanical characteristics of the used NM have a strong influence: The thickness is one possible factor that orchestrates the interplay of PRF and the respective membrane mechanistically by impeding material-cell interaction. In comparison with the material used in other studies, NM is thicker (1 mm of the NM vs. 0.15 mm of the jason® and 0.4 mm of the collprotect® membrane (all: botiss biomaterials GmbH, Zossen, Germany)). The fact that a membrane with similar thickness (1.2–1.7 mm, Mucoderm (botiss biomaterials GmbH, Zossen, Germany)) demonstrates comparable data strengthens this hypothesis [26].

This is in accordance with the immunohistochemical findings provided in this study: Although an interaction between NM and PRF could be quantified via elevated vessel formation in the CD105 staining after 24 h, this effect leveled over time. This shows that the initially enhanced branching points help to exploit the membrane with newly formed vessels, but the pro-angiogenic effect is not longstanding. This is explainable inter alia by the mentioned mechanical characteristics of the membrane, especially the thickness and specific collagen network seem to orchestrate interaction. Solid PRF alone therefore exhibited the most vessels in the immunohistochemical staining; this pro-angiogenic potential was already proven in past studies [26].

Al-Maawi et al. provided comprehensive data on how injectable PRF interacts with different collagen matrices in detail, with significant differences in penetration and absorption mechanism depending on the respective collagen biomaterial [41]. The weak additional pro-angiogenic characteristics achieved by pre-vascularization of NM with injectable PRF may, therefore, be explained by a type I or II interaction type with no or partial penetration. This may explain that stable PRF alone enhanced neo-vessel formation after 24 h compared to NM in combination with liquid PRF. There is no evidence of a detailed interaction of stable PRF and collagen matrices; future studies must analyze the distinguished penetration and adsorption mechanism. From the data of this study, no mechanistic differences between liquid and solid PRF for pre-vascularization of collagen matrices could be obtained.

As a further explanation of the data, the pro-angiogenic potential heavily relies on the PRF membrane’s cell content and growth factor release kinetics. Here, the injectable PRF has the highest concentration of leukocytes and platelets compared to the solid PRF membrane [17, 18]. In the solid version, more neutrophilic granulocytes were detected, especially at the interface between erythrocytes and the buffer layer [24]. However, there needs to be more consistent evidence about the differences in the growth factor extent of the respective formulas. In a preclinical study, Zwittnig et al. found little variations between solid and liquid PRF regarding growth factor release [42]. In contrast, Shah et al. detected solid PRF superior to injectable PRF in terms of mechanical strength and increased growth factor releases of TGF-b, PDGF-BB, and VEGF. This enhanced cell viability, alkaline phosphatase production, and mineralization in human periodontal ligament cells [43]. Concerning the presented results, there was no significant difference in vascularisation when the membrane was biofunctionalized using solid or injectable PRF. However, growth factor release kinetics were not addressed as a further study limitation. To conclude, there is no sufficient evidence to support either the liquid or solid PRF regarding “biological activity” as the material of choice in the pre-vascularization of collagen matrices.

This study suffers from some limitations. First, it is a preclinical experimental study to evaluate distinctive interactions between the membrane and the respective APC. Furthermore, a short observation period was chosen since the focus was the early interaction. So far, multiple other groups have shown the described pro-angiogenic effects in the clinical setting [44–46]. The presented pre-clinical supplementary data should support clinicians in day-to-day decisions in dentoalveolar surgery.

## Conclusions

The results in the present study provide a further understanding of the biofunctionalization of different collagen membranes with PRF concerning their rehydration and vascularization. Using injectable PRF for rehydration protocol of the collagen membrane seems superior to saline solution, leading to acidosis that can ultimately optimize wound healing. Additionally, this study demonstrated for the first time that an acellular collagen membrane of porcine origin can display high pro-angiogenic characteristics that could not exaggerate with pre-vascularization with PRF either in solid or in liquid form. The results indicate that more evidence is needed in the direct physio-mechanical interplay and adjacent growth factor release of different collagen matrices and autologous platelet concentrates such as PRF. With this evidence, algorithms for clinical practice in reconstructive surgery can be generated which membrane can be used in the respective clinical setting and if pre-vascularization with APC can optimize outcomes.

## Data Availability

Not applicable.
